# Sex differences in cortical volume and gyrification in autism

**DOI:** 10.1186/s13229-015-0035-y

**Published:** 2015-07-04

**Authors:** Marie Schaer, John Kochalka, Aarthi Padmanabhan, Kaustubh Supekar, Vinod Menon

**Affiliations:** Department of Psychiatry and Behavioral Sciences, Stanford University School of Medicine, Stanford, CA 94305 USA; Program in Neuroscience, Stanford University School of Medicine, Stanford, CA 94305 USA; Department of Neurology and Neurological Sciences, Stanford University School of Medicine, Stanford, USA

**Keywords:** Gyrification, Females, Cortical volume, Neuroimaging, Cerebral morphometry, Sex differences

## Abstract

**Background:**

Male predominance is a prominent feature of autism spectrum disorders (ASD), with a reported male to female ratio of 4:1. Because of the overwhelming focus on males, little is known about the neuroanatomical basis of sex differences in ASD. Investigations of sex differences with adequate sample sizes are critical for improving our understanding of the biological mechanisms underlying ASD in females.

**Methods:**

We leveraged the open-access autism brain imaging data exchange (ABIDE) dataset to obtain structural brain imaging data from 53 females with ASD, who were matched with equivalent samples of males with ASD, and their typically developing (TD) male and female peers. Brain images were processed with FreeSurfer to assess three key features of local cortical morphometry: volume, thickness, and gyrification. A whole-brain approach was used to identify significant effects of sex, diagnosis, and sex-by-diagnosis interaction, using a stringent threshold of *p* < 0.01 to control for false positives. Stability and power analyses were conducted to guide future research on sex differences in ASD.

**Results:**

We detected a main effect of sex in the bilateral superior temporal cortex, driven by greater cortical volume in females compared to males in both the ASD and TD groups. Sex-by-diagnosis interaction was detected in the gyrification of the ventromedial/orbitofrontal prefrontal cortex (vmPFC/OFC). Post-hoc analyses revealed that sex-by-diagnosis interaction was driven by reduced vmPFC/OFC gyrification in males with ASD, compared to females with ASD as well as TD males and females. Finally, stability analyses demonstrated a dramatic drop in the likelihood of observing significant clusters as the sample size decreased, suggesting that previous studies have been largely underpowered. For instance, with a sample of 30 females with ASD (total *n* = 120), a significant sex-by-diagnosis interaction was only detected in 50 % of the simulated subsamples.

**Conclusions:**

Our results demonstrate that some features of typical sex differences are preserved in the brain of individuals with ASD, while others are not. Sex differences in ASD are associated with cortical regions involved in language and social function, two domains of deficits in the disorder. Stability analyses provide novel quantitative insights into why smaller samples may have previously failed to detect sex differences.

**Electronic supplementary material:**

The online version of this article (doi:10.1186/s13229-015-0035-y) contains supplementary material, which is available to authorized users.

## Background

Autism spectrum disorder (ASD) is characterized by impaired social interactions, altered communication skills, and restricted interests or repetitive behaviors [1]. Recent estimates suggest that ASD affects one in 68 children in the US [2], with a strong sex-related bias. On average, the male to female ratio is estimated to be 4.3:1 [3]. This striking sex difference raises important questions regarding potential protective factors in females [4, 5]. For instance, the extreme male brain theory of autism [6] links ASD etiology to the masaculinizing effects of fetal testosterone [7] and postulates that ASD represents an exaggerated expression of such masculinization. An alternate hypothesis is that activation of the maternal immune system following infection may have a sex-specific effect on the developing fetal brain, targeting microglia [8] and increasing the risk of ASD [9]. However, these theories remain controversial and progress in the field has been hampered by the limited number of neurobiological studies on sex differences in affected individuals.

Little is known about neuroanatomical differences in ASD between males and females, because most studies of individuals with ASD are based on small samples with limited numbers of females. An important question is whether there are sex differences in brain structures associated with the core phenotypes of the disorder: language, social communication, and repetitive and restricted behaviors and interests (RRBI). To the best of our knowledge, only four studies to date have specifically assessed sex differences in cortical morphometry of individuals with ASD. Initial studies included fewer than 10 females with ASD [10, 11]. First, Bloss et al. [10] reported that girls with ASD show more anomalies in cerebral lobes volumes than affected boys. Two years later, Schumann et al. [11] similarly reported more severe structural anomalies in the amygdala in girls with ASD. More recent studies have examined local differences in gray and white matter in larger samples of individuals using voxel-based morphometry (VBM) [12, 13]. In a sample of 58 participants (*n* = 13 females with ASD), Beacher et al. [12] observed a significant sex-by-diagnosis interaction in the right inferior parietal lobe, with ASD individuals showing an attenuation of the typical male > female volumetric difference. The largest study to date examined 120 participants (*n* = 30 females with ASD) [13]. Using VBM, Lai and colleagues did not find any significant sex-by-diagnosis interactions in gray matter volume but reported several clusters of sex differences in white matter volume. Not surprisingly, none of the results published to date have been replicated, underscoring the substantial heterogeneity of the ASD phenotype [14–16] and highlighting the need for samples with larger numbers of females.

Previous studies have been based on manual delineation of the amygdala [11], semi-automated extraction of lobar volumes [10], or voxel-based morphometry [12, 13]. Importantly, none of these studies have used surface-based morphometry to provide a comprehensive characterization of sex differences in structural measures such as cortical volume, thickness, and gyrification. Compared to volume-based methods, surface-based methods more accurately reflect the cortical geometry and have proven to be more powerful and reliable in detecting effects, with fewer subjects required to achieve similar levels of significance [17, 18]. Surface-based methods also allow the distinction between cortical thickness [19] and gyrification [20], which provide complementary information about the timing and nature of disrupted neurodevelopmental processes (reviewed in [21]). Crucially, altered gyrification is thought to reflect early cortical development [22–25], whereas altered cortical thickness is associated with later cortical maturation during childhood and adolescence [26, 27]. To complement these specific measures, surface-based measurements of local cortical volume can also be used to provide more direct comparison to results from previous studies using voxel-based morphometry.

Here, we leverage a new, large, open-access dataset [28] to investigate sex differences in brain structure in ASD, using surface-based morphometry. This dataset (autism brain imaging data exchange (ABIDE)) is a consortium effort between 17 international sites sharing their neuroimaging data and collectively contains the largest sample of females with ASD available to date. After careful quality control, we matched each female with ASD (*n* = 53) for age and site with one male with ASD. We also matched typically developing (TD) males and females using the same procedures. We then used surface-based morphometry to measure local cortical volume, thickness, and gyrification at each of ~150,000 vertices per hemisphere. A whole-brain approach with stringent correction for multiple comparisons was used to assess the main effect of sex, main effect of diagnosis, and sex-by-diagnosis interaction. Finally, we used bootstrap procedures to examine the stability of our findings and carried out post-hoc power analyses based on the observed effect sizes to estimate the relationship between observed statistical power and sample size.

Sex differences in the brains of typically developing individuals have been extensively examined over the last two decades, with a number of studies reporting robust differences between males and females [29–31]. Based on these extant studies and generally weak volume-based morphometric differences between TD and ASD groups [14–16], we expected significant main effects of sex in the combined group of TD and ASD participants. Preserved typical sex differences in ASD would suggest that the factors underlying the etiology of ASD are, at least partially, independent of the sex. However, genetic [32–35], biochemical [36], and animal [37, 38] studies have provided robust evidence for sex-specific biomarkers of autism, lending support to the idea that males and females with ASD may present different clinical and neuroanatomical phenotypes. Clinical studies thus far have reported mixed findings regarding potential differences in the clinical and cognitive profiles of males and females with ASD [9, 39]. In the absence of clear phenotypic differences and given the paucity of neuroanatomical studies of sex differences in ASD published to date, it was challenging to generate a priori hypotheses regarding which cortical regions would show significant sex-by-diagnosis interactions. Nevertheless, we expected to observe a significant interaction in cortical areas known to play a role in autistic symptoms, such as “social brain” areas as a substrate for social and communication difficulties [40, 41], cortical nodes of the saliency network, which might be responsible for difficulties in integrating external sensory stimuli and internal states [42, 43], and/or pre-motor/motor areas densely connected with the striatum as a substrate for repetitive behaviors [44, 45].

## Methods

### Participants

Demographic, cognitive assessment, and structural MRI data from 539 individuals with ASD and 573 typical controls (age 6–56 years old), acquired across 17 international sites, were obtained from the open-access ABIDE database [28]. For each of these sites, approval of the study protocol by the Institutional Review Board or an explicit waiver to provide fully anonymized data, was required by the ABIDE Consortium before data contribution [28]. A comprehensive list of all the review boards that approved the study is provided in the “Acknowledgements” section. Further, in accordance with Health Insurance Portability and Accountability (HIPAA) guidelines, the ABIDE Consortium ensured that all the datasets were fully anonymized, with no protected health information included. Diagnosis of ASD was performed using the Autism Diagnostic Observation Schedule (ADOS, [46]), the Autism Diagnostic Interview—Revised (ADI-R, [47]), or both. Given the low prevalence of females with ASD, this large-scale dataset represents a unique opportunity to study a large sample of females with ASD. However, this unprecedented advantage comes at the expense of the need for combining MRI data from different sites, using different acquisition parameters and yielding different data quality. To overcome this limitation, we used careful individual matching for site and age. These procedures are described below.

We first examined and processed the cerebral T1-weighted MRI acquisitions for all the 1112 participants, obtaining accurate three-dimensional cortical models for 945 participants (see details in the “Image Processing” section below). Exclusion criteria included apparent motion artifact, sub-optimal contrast impairing the tissue segmentation, or incompleteness of the structural acquisition. We then selected all females with ASD. The resulting 53 females with ASD were individually matched for site and for age with 53 males with ASD. Similarly, the 53 females with ASD were individually matched for site and for age with TD females and then TD males. Unfortunately, two sites were missing a matching TD female, so our final sample contained 51 TD females and 53 TD males. Data from the 210 selected individuals was collected at 11 sites (Caltech: *n* = 16; CMU: *n* = 12; KKI: *n* = 16; Leuven: *n* = 8; Max_Mun: *n* = 12; NYU: *n* = 40; OLIN: *n* = 11; Pitt: *n* = 16; UCLA: *n* = 23; UM: *n* = 24; Yale: *n* = 32; age range 8.1–39.3 years old). A detailed description of the final sample examined in the present study is provided in Table 1; the matching procedure is described in detail in Additional file 1: Table S1. All 106 patients included in the present study had a clinical ASD diagnosis. Of the 91 for which the ADOS-G [46] or revised ADOS Gotham [48] scores were available, 5 did not meet the ASD criteria at the ADOS: an 18 year old female had an ADOS total that was 4 points below the cut-off, a 10 year old female had an ADOS Gotham score that was 3 points below the cut-off, and 3 additional patients did not meet the cut-off by one point for either the ADOS-G or the ADOS Gotham scores (1 male, 2 females). These 5 patients were however largely above the cut-off scores for autism as measured by the ADI-R [47] (social domain 13–24; communication domain 11–19; RRB 5–12; onset of the anomalies 2–5).

**Table 1:** Description of the study sample

	ASD F	ASD M	TD F	TD M	*p*	*p*_ASDF-ASDM_	*p*_ASDF-TDF_	*p*_TD-TDM_	*p*_ASDM-TDM_	*p*_ASDF-TDM_	*p*_ASDM-TDF_
Age	17.1 ± 8.3	17.2 ± 8.4	17.2 ± 7.6	17.1 ± 8.2	>.999	>.999	>.999	>.999	>.999	>.999	>.999
Cognitive variables											
FSIQ	107.8 ± 17.1	102.4 ± 16.5	111.2 ± 12.1	107.8 ± 9.7	0.024	0.318	0.702	0.696	0.316	>.999	0.028
VIQ	106.4 ± 19.2	103.7 ± 15.7	110.7 ± 13.3	107.2 ± 11.2	0.207	0.880	0.633	0.776	0.761	0.995	0.215
PIQ	103.7 ± 16.8	102.0 ± 15.9	107.3 ± 12.7	106.2 ± 10.8	0.294	0.958	0.712	0.989	0.574	0.869	0.396
Autistic symptoms											
ADOS total	11.3 ± 4.0	11.9 ± 3.9				0.533					
ADOS social	7.7 ± 2.8	8.0 ± 2.9				0.663					
ADOS comm.	3.7 ± 1.6	4.0 ± 1.4				0.409					
ADOS RRB	2.1 ± 1.5	1.9 ± 1.3				0.591					
ADI social	19.8 ± 5.9	20.8 ± 5.3				0.441					
ADI comm.	15.5 ± 5.0	16.0 ± 4.3				0.683					
ADI RRBI	5.8 ± 2.3	5.8 ± 3.0				0.896					

As a result of missing information in the ABIDE dataset, the cognitive and clinical descriptions encompass the following sample sizes: FSIQ: *n* = 50 ASD F, 50 ASD M, 48 TD F, 51 TD M; VIQ: *n* = 43 ASD F, 43 ASD M, 42 TD F, 43 TD M; PIQ: *n* = 43 ASD F, 44 ASD M, 41 TD F, 45 TD M; ADOS (total, social, communication): *n* = 33 ASD F, 33 ASD M; ADOS stereotyped behaviors: *n* = 30 ASD F, 30 ASD M; ADI social, communication and RRBI: *n* = 38 ASD F, 42 ASD M

### Image processing

MRI images were processed with FreeSurfer (http://surfer.nmr.mgh.harvard.edu, version 5.3) in each individual’s native space. Prior to cortical reconstruction, all images were resampled to a common isotropic voxel size of 1 × 1 × 1 mm. For each participant, cortical reconstructions were carefully inspected on a slice-by-slice basis and corrected by a single experienced FreeSurfer user (MS). Following thorough quality control, 15 % of the acquisitions from the original ABIDE sample had to be excluded, mainly due to excessive head motion. Among the remaining good quality acquisitions (*n* = 945), the samples were selected via the matching process detailed above. Cerebral volumes were extracted using previously described procedures [49]. Briefly, the processing steps involved (1) removing non-brain tissue, (2) executing automatic segmentation of the subcortical gray matter structures, and (3) extracting cortical surfaces [50, 51]. Both intensity and continuity data from the entire three-dimensional MR volume were used in the segmentation procedures, thus producing accurate representations of cortical thickness and volume. These procedures have been validated against histological studies [52] and have been shown to be reliable across scanner models and field strengths [53]. The reconstruction process resulted in measurements for cortical volume, cerebral white matter volume, and subcortical volume. Supratentorial volume was also computed as the sum of cortical, cerebral white matter, and subcortical volumes. Intracranial volume was not extracted, as some sites did not include the entire cerebellum in their field of view.

Vertex-wise measurements of cortical volume and thickness were computed from the three-dimensional cortical mesh models at more than 150,000 points over each hemisphere in native space [19]. Finally, local gyrification index (*l*GI) was measured at each point using previously validated algorithms [20]. *l*GI is a surface-based measure of the degree of cortical folding that quantifies the amount of cortex buried within the sulcal folds in the surrounding circular region. Inter-subject comparisons of the cortical volume, thickness, and *l*GI values were performed through spherical registration of the surfaces to the *fsaverage* template space, a transformation that minimizes metric distortion and allows for a highly reliable point-to-point comparison of cortical measures between groups [54, 55]. Cortical volume and thickness maps were smoothed using a 10 mm full width at half maximum (FWHM) two-dimensional Gaussian kernel (yielding an overall degree of smoothness of 14.5 and 14.4 mm for volume and of 17.0 and 16.7 mm for thickness values, for the left and right hemisphere, respectively). As *l*GI measure is already intrinsically smooth, the data were only minimally smoothed (1 mm FWHM) to achieve a similar level of smoothness to the cortical volume and thickness data (final degree of smoothness in the *l*GI data was 16.3 and 15.9 mm for the left and right hemisphere respectively).

### Statistical analyses

**Fig. 1:**
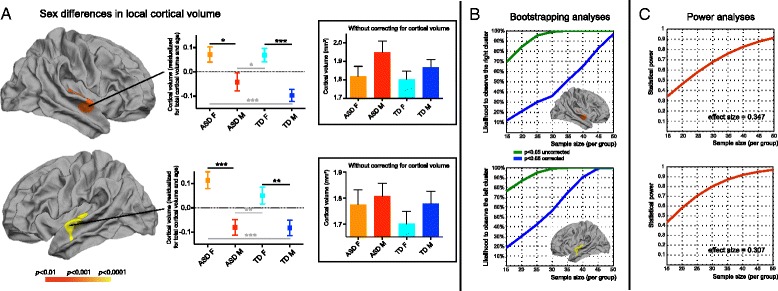
Main effect of sex: local cortical volume. **a** Whole-brain analyses (*p* < 0.01, corrected). In each hemisphere, one cluster at the pSTG/PT showed a relative increase in cortical volume in females compared to males, both within each diagnostic group (*black signs*) and across diagnostic groups (*gray signs*). For the post-hoc two-by-two analyses, the following *p* values are depicted: *p < 0.05, **p < 0.01, ***p < 0.001. **b** Bootstrapping analyses. The likelihood to observe both clusters was tested using a bootstrap procedure, simulating sample sizes ranging from 15 to 50 individuals in each group (total *n* = 60–200). For a sample size of 30 females with ASD, the probability to observe the pSTG/PT clusters at the level of *p* < 0.05 (corrected) was below 40 % for the right hemisphere and below 60 % for the left hemisphere. **c** Power analyses. Plot depicting the relationship between statistical power and sample size, computed a posteriori based on the effect sizes obtain in the full dataset

**Fig. 2:**
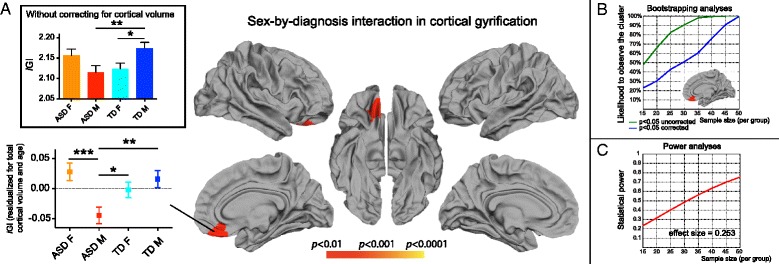
Sex-by-diagnosis interaction: local gyrification. **a** Whole-brain analyses (*p* < 0.01, corrected). In this vmPFC/OFC cluster, we found a significant sex-by-diagnosis interaction, males with ASD show a selective reduction in gyrification as compared to all three other groups. When the smaller brain size of females is not taken into account (*upper panel*), TD females typically show reduced gyrification. This is however not the case within the ASD group, where even raw *l*GI values tend to be higher in females than males with ASD. For the post-hoc two-by-two analyses, the following *p* values are depicted: *p < 0.05, **p < 0.01, ***p < 0.001. **b** Bootstrapping analyses. The likelihood to observe a significant sex-by-diagnosis interaction in the vmPFC/OFC cluster was tested using a similar bootstrap procedure as for the main effect of sex. Again, the pattern of a decrease in the likelihood to observe any significant effect as the sample size decreases suggests that previous studies of sex differences in ASD were likely underpowered to estimate robust and reproducible effects. **c** Power analyses. Plot depicting the relationship between statistical power and sample size, computed a posteriori based on the effect sizes obtain in the full dataset

We used a general linear model (GLM) to estimate the effect of sex, diagnosis, and sex-by-diagnosis interactions on all neuroanatomical variables, including age as a covariate. Cortical volume was also included as a covariate in the analyses of local cortical volume and gyrification to account for sex-related differences in brain scaling (see Table 2). Given that mean cortical thickness did not differ between males and females, we did not include any additional covariate in the cortical thickness analysis (see Results). A statistical threshold of *p* < 0.01 (corrected for multiple comparisons using Monte Carlo simulations [56]) was used for all analyses, to provide stringent criteria to minimize false positives. Clusters with significant effects of diagnosis, sex, or sex-by-diagnosis interactions were further tested using two-by-two analysis of covariance (ANCOVA) analyses between the four groups. For the post-hoc analyses, a more permissive significance threshold is reported on the plots, with the following *p* values provided in Figs. 1 and 2: *p < 0.05, **p < 0.01, ***p < 0.001.

**Table 2:** Brain volumes in the four groups

	ASD F	ASD M	TD F	TD M	*p*_diag_	*p*_sex_	*p*_sex-by-diag_
Supratentorial	982 ± 120	1,109 ± 124	981 ± 91	1,098 ± 105	0.695	<0.001	0.743
Cortical	493 ± 71	554 ± 72	490 ± 56	547 ± 66	0.561 (0.607)	<0.001 (0.353)	0.826 (0.869)
Cerebral white	413 ± 62	470 ± 68	416 ± 49	470 ± 68	0.836 (0.279)	<0.001 (0.571)	0.904 (0.746)
Subcortical	60.2 ± 5.0	65.6 ± 5.3	59.9 ± 4.9	64.6 ± 5.1	0.352 (0.306)	<0.001 (0.224)	0.604 (0.671)

All values are in cubic centimeters and correspond to the sum of the corresponding values for the left and right hemisphere. The *p* values in parenthesis denote significance when the supratentorial volume is additionally included in the model

To evaluate the robustness of our findings with respect to sample size and to sampling bias related to inter-individual differences, we carried out a bootstrap analysis building on the individual matching described above and in Additional file 1: Table S1. In this procedure, age-, site-, and sex-matched “quartets” made of one individual from each group were randomly subsampled from the full cohort without replacement. For each new subsample, vertex-wise statistical analyses similar to those above were conducted to examine a significant sex-by-diagnosis interaction, using a statistical threshold most commonly used in structural imaging studies (*p* < 0.05, corrected and uncorrected). We simulated sample sizes ranging from 15 to 50 individuals in each group (with steps of 5), using 500 bootstrapped subsamples for each sample size. The procedure was used to estimate the likelihood of finding the “true” result we observed in the full cohort from smaller samples.

## Results

### Demographic and cognitive profile

As detailed in Table 1, the four groups (females with ASD, males with ASD, TD females, TD males) did not differ in age (*F* = 0.002, *p* > 0.999), verbal IQ (*F* = 1.536, *p* = 0.207), or performance IQ (*F* = 1.248, *p* = 0.294). However, the four groups differed in full scale IQ (*F* = 3.2, *p* = 0.024), driven by higher full scale IQ in TD females compared to males with ASD (*p* = 0.028).

### Autism symptoms

Males and females with ASD did not differ in overall severity of autism as measured with total scores at the ADOS (*F* = 0.393, *p* = 0.533). There were also no sex differences in social and communication deficits as well as restricted and repetitive behavior, as measured with ADOS and ADI-R (all *ps* ≥ 0.441).

### Brain volumes

A series of 2 × 2 ANCOVA analyses, with supratentorial, cortical, white matter, and subcortical volumes as dependent variables, sex and diagnosis as fixed factors, and age as a covariate revealed a significant main effect of sex on all these measures (all *ps* < 0.001) but not diagnosis (all *ps* ≥ 0.352) or their interaction (all *ps* ≥ 0.507). The main effect of sex was driven by significantly smaller supratentorial (*F* = 63.559, *p* < 0.001), cortical (*F* = 44.379, *p* < 0.001), white matter (*F* = 52.145, *p* < 0.001), and subcortical (*F* = 51.954, *p* < 0.001) volumes in females, compared to males (Table 2). After correcting for differences in the supratentorial volume, sex differences in the cortical (*p* = 0.353), white matter (*p* = 0.571), and subcortical (*p* = 0.224) volumes were not significant, suggesting that the observed sex-related differences were driven by differences in supratentorial volume. To account for the observed sex-related scaling in brain volumes, we corrected for cortical volume in subsequent analyses of local cortical volume and gyrification.

To assess whether cortical thickness measures also had to be corrected for a sex-related scaling effect, we conducted 2 × 2 ANCOVA analyses on mean cortical thickness for each hemisphere, which did not reveal any significant main effects of sex (left: *p* = 0.221, *F* = 1.510; right: *p* = 0.152, *F* = 2.069), diagnosis (left: *p* = 0.355, *F* = 0.858; right: *p* = 0.596, *F* = 0.281), or their interaction (left: *p* = 0.649, *F* = 0.207; right: *p* = 0.852, *F* = 0.035). We therefore did not introduce any additional covariates in the cortical thickness analyses.

### Local cortical volume, thickness, and gyrification

Vertex-wise analyses of local cortical volume showed a significant main effect of sex, but not diagnosis or their interaction. The main effect of sex was observed in homologous regions of the posterior superior temporal cortex (pSTG) extending to the planum temporale (PT). For both clusters, we verified that the main effect of sex remained significant after including site as a covariate in the model. We also verified that the results were not altered when taking full scale intelligence quotient (FSIQ) into account. Females had larger cortical volumes in the right (5.95 cm^2^, cluster-wise *p* = 0.003, effect size = 0.347) as well as the left pSTG/PT clusters (8.3 cm^2^, cluster-wise *p* = 0.0001, effect size = 0.307). Post-hoc analyses demonstrated that the greater pSTG/PT cortical volume in females compared to males was significant in ASD (left: *F* = 21.934, *p* < 0.001; right: *F* = 10.315, *p* = 0.002) as well as TD (left: *F* = 8.981, *p* = 0.003; right: *F* = 15.561, *p* < 0.001) groups (Fig. 1a).

Cortical thickness analyses revealed no significant effects of sex, diagnosis, or sex-by-diagnosis interaction.

For local cortical gyrification (*l*GI), there was a significant sex-by-diagnosis interaction, but no main effects of sex or diagnosis. The significant sex-by-diagnosis interaction was observed in one cluster that extended from the right ventromedial prefrontal cortex (vmPFC) to the orbitofrontal cortex (OFC) (7.99 cm^2^, cluster-wise *p* = 0.004, effect size = 0.253; see Fig. 2a). We verified that the sex-by-diagnosis interaction remained significant in this cluster after including site as a covariate in the model. We also verified that the results remained unchanged when taking FSIQ into account. Post-hoc analysis revealed that among the four groups examined, after correction for volumetric differences, males with ASD had the lowest *l*GI while females with ASD had the highest *l*GI values, in the vmPFC/OFC.

To further assess the relevance of this vmPFC/OFC cluster, we performed additional whole-brain analyses to examine the effect of sex within the ASD group (ASD M *vs* ASD F), and the effect of diagnosis within each sex group (ASD M *vs* TD M; and ASD F *vs* TD F) on vertex-wise measurements of gyrification. Results of these whole-brain analyses were consistent with the results from the vertex-wise analysis, namely, males with ASD, compared to females with ASD, had lower *l*GI within a vertex-level cluster encompassing the vmPFC/OFC regions (19.23 cm^2^, cluster-wise *p* = 0.0001) (see Additional file 2: Figure S1a). Additionally, another cluster showed lower *l*GI in the homologous left OFC region in males with ASD compared to females with ASD (6.95 cm^2^, cluster-wise *p* = 0.0016). Furthermore, consistent with the results of the vertex-wise *l*GI analysis, a cluster spanning the vmPFC/OFC showed lower gyrification in males with ASD, compared to TD males (7.04 cm^2^, cluster-wise *p* = 0.0022; see Additional file 2: Figure S1b). No cortical regions exhibited lower or higher gyrification in females with ASD compared to TD females (see Additional file 2: Figure S1c).

Taken together, our results from local gyrification analysis point to atypical sex differences in the vmPFC/OFC, mainly driven by a *l*GI reduction in males with ASD.

### Stability analyses

Given the large individual differences in the ASD phenotype, we next investigated whether the observed clusters of sex differences in cerebral morphometry depend on sample size. For this purpose, we performed a bootstrap procedure, sampling different sized subsets from the full cohort of 210 participants. We explored sample sizes ranging from 15 to 50 individuals from each of the four groups, generating 500 random samples for each. With each sample, vertex-wise statistical analyses similar to those above were performed, to assess the significance of main effect of sex on cortical volume and sex-by-diagnosis interaction on local gyrification. These simulation analyses used a statistical threshold of *p* < 0.05, and results were aggregated across subsamples. The result of this bootstrap subsampling analysis yielded a cortical map with a frequency of observing a significant result for each vertex for each simulated sample size.

Analysis of all the individual cortical maps for the sex-by-diagnosis interaction revealed that, except the vertices in the right vmPFC/OFC region, no other cortical vertex survived statistical threshold (*p* < 0.05, corrected) in more than 10 % of the simulated samples, for any of the sample sizes studied (15 to 50, in increments of 5). For the main effect of sex, only one other small cluster in addition to the vertices in the bilateral STG region showed significance at *p* < 0.05 (corrected) in more than 10 % of simulated subsamples: a cluster in the right occipital pole showed a significant effect of sex in 12.4, 15.4, and 29 % of the simulated subsamples for sample sizes of 40, 45, and 50 participants respectively in each of the four groups. Of note, this small cluster was not significant in the final sample size of 210 participants, even at the threshold of *p* < 0.05 (corrected).

We next examined the effect of sample size in the clusters that demonstrated a significant main effect of sex and sex-by-diagnosis interaction in the full dataset. For both the main effect of sex and the sex-by-diagnosis interaction, a dramatic drop in the likelihood to detect these significant clusters was observed as the sample size decreased (Figs. 1b and 2b). For instance, with 30 individuals per group (total *n* = 120), the vmPFC/OFC cluster that showed a significant sex-by-diagnosis interaction in the full sample survived statistical correction in only ~50 % of the bootstrapped subsamples (Fig. 2b). For the same sample size, bootstrapping analyses revealed that the main effect of sex in the bilateral pSTG/PT clusters was found in less than 60 and 40 % of the subsamples respectively (Fig. 2b). These stability analyses demonstrate that a sufficiently large sample size is required to identify the effects we report.

### Post-hoc power analyses

Finally, we conducted a posteriori power analyses using published algorithms [57] to compute the achieved power based on the sample size and effect size. These analyses yielded a power of 0.779 for the sex-by-diagnosis interaction in the right vmPFC/OFC, 0.930 for the left pSTG/PT cluster that showed a main effect of sex, and 0.977 for its right counterpart. The relationship between achieved power and sample size for the effect sizes observed in this study is plotted in Figs. 1c and 2c. Given an effect size of 0.253, achieving a power of 0.8 in the cluster of significant sex-by-diagnosis interaction would require a few more individuals than we had available: 55 individuals per group. For the main effect of sex however, power analyses revealed that the left cluster would reach a power of 0.8 with ~38 individuals per group (total *n* = 151), and the right cluster with 30 individuals per group (total *n* = 120). As only one study to date achieved a sample of 30 females with ASD with a total sample size of 120, the result of the stability and power analyses suggests that all previous studies examining sex differences in cerebral morphometry in ASD were underpowered.

## Discussion

To our knowledge, this study is the largest of its kind to examine sex differences in brain anatomy in ASD. We used a surface-based morphometric approach for a more accurate characterization of the location and nature of anatomical differences between the ASD and TD groups, focusing on both main effects of sex which identifies common brain areas that show sex differences, as well as sex-by-diagnosis interaction which identifies brain areas where the two groups diverge in their pattern of sex differences. We found that some aspects of typical sex differences in brain structure are preserved in ASD, while others are not. We identified clusters of preserved and altered sex differences encompassing cortical regions involved in language and social communication, two core processes affected in the disorder. The anatomical localization of these clusters might help shed light on neurobiological mechanisms leading to autistic symptoms that are shared in males and females, as well as those that are sex-specific.

Neurotypical sex differences, characterized by larger volume in females compared to males in the posterior superior temporal cortex/planum temporale (pSTG/PT), were preserved in individuals with ASD. This suggests that sex differences in language acquisition [58, 59] and language processing [60–63] systems of the brain that are commonly reported in typically developing individuals might also be observed in individuals with autism. Preserved sex differences in cortical areas responsible for language processing also suggest that the mechanisms leading to language difficulties in ASD do not differ between affected males and females. In contrast, atypical sex-specific alterations of gyrification patterns were found in the orbitofrontal/ventromedial prefrontal cortex in individuals with ASD, with reduced gyrification observed in affected males only. Based on previous reports that *l*GI changes little with development [64], this pattern of altered gyrification points to early abnormal development of the orbitofrontal/ventromedial prefrontal cortex in males with ASD and provides a sex-specific biological substrate in a cortical region that forms part of the “social brain” [65, 40].

### Preserved sex differences in the temporal cortex in ASD

A main effect of sex was observed in both groups, with larger pSTG/PT cortical volume in females relative to males. In these clusters, we did not observe any significant sex-by-diagnosis interaction, rather we found a similar pattern of relative volumetric increase in both TD females and females with ASD compared to TD males and males with ASD. This finding suggests that one of the brain structures that is most typically related to a core symptom of ASD, namely language difficulties, does not show any sex-specific differences.

In typically developing individuals, many studies have reported a relatively enlarged superior temporal gyrus (STG) in females compared to males [66–69]. A large body of literature relates sex differences in the anatomy and function of perisylvian structures (inferior frontal and superior temporal cortex, along with planum temporale) to differences in various aspects of language processing, including semantic [61], phonological [63, 62], and narrative processing [60]. In addition to these functional processing differences, language acquisition appears to follow a sex-specific trajectory: typically developing girls have been shown to mature more rapidly than boys in specific language skills including early communicative gestures, and expressive language [58]. These sex differences have been shown to persist until later in childhood [59] but tend to fade by adulthood [70].

In individuals with ASD, studies including predominantly or only males have shown differences in STG structure [71, 72] and in STG activation during speech processing [73]. Inter-individual variations in STG volume [71] and in trajectories of volumetric STG growth [72] were further related to history of language delay in males with ASD. Future studies are required to better understand the functional significance of preserved sex differences in the STG. In the meantime, based on extant functional imaging data, we suggest that our findings may reflect sex differences in language acquisition and processing in individuals with ASD. Although quantitative studies measuring sex differences in language and communication skills have been inconsistent so far [74–76], some clinical studies suggest that girls with ASD acquire language skills faster than their male peers. For instance, girls with higher IQ tend to be diagnosed later than males, as their superior language skills during their first years of life might mask autistic symptoms [77, 78, 74]. Also, a study using retrospective parent reports has suggested that, at the age of 4 years old, girls with ASD present less severe communication difficulties compared to affected boys [79]. These studies suggest that the sex differences in language and communication seen in typical development, with faster acquisition of language and better communication skills in girls, might be relatively preserved in ASD. Future studies including more fine-grained measures of language skills and history of language development are required to better understand if preserved sex differences in pSTG/PT cortical volumes relates to sex-related differences in trajectories of language acquisition in young children with ASD.

### Altered sex differences in the ventromedial/orbitofrontal cortex in ASD

We detected one robust cluster of atypical sex differences in ASD, characterized by a reduction in local gyrification of the right vmPFC/OFC region in males with ASD compared to the other three groups (Fig. 2 and Additional file 2: Figure S1). Altered sulcal patterns of the OFC region in males with ASD are consistent with the recent study by Watanabe and colleagues [80], who observed differences in the distribution of the orbitofrontal sulcal subtypes in high-functioning adult males with ASD compared to TD. The vmPFC/OFC region is known to play a role in mentalizing and self-reflection [81], affective theory of mind [82], emotion recognition [83], and social motivation [84]. Also, a recent eye-tracking study suggests that the vmPFC/OFC region is critical in identifying socially salient stimuli [85]: patients who underwent neurosurgery for vmPFC/OFC lesions demonstrate decreased fixation to the eyes region. A selective disruption of the vmPFC/OFC cortical structure in males might provide a biological substrate for reduced processing of social saliency [86, 87].

Functional neuroimaging studies, predominately with male participants, have shown altered function of the vmPFC/OFC region. First, task-based fMRI studies have reported altered activity of the vmPFC/OFC in predominantly male samples during theory of mind [88] and reward processing [89] including social reward [90], tasks. Second, resting state fMRI studies have identified altered connectivity in anterior nodes of the default-mode network [91–93, 40], which overlap with the vmPFC/OFC cluster identified in this study. Finally, oxytocin administration has been shown to increase both OFC activity and orientation to social stimuli in a sample composed of 18 boys and 3 girls with ASD [94]. The fact that all these studies were conducted in samples composed mostly or entirely of affected males suggests altered function of the vmPFC/OFC in males with ASD but leaves the question of potential functional alterations of this region in affected females unanswered. Further research is needed to investigate whether the function of the vmPFC/OFC differs in females with ASD. Extant reports suggest that females with ASD have a greater desire to interact with others, tend to imitate their peers more, and develop better compensatory strategies to mask their difficulties relative to males (reviewed in [9]). Taken together, our findings of a sex-specific disruption of cortical development in the vmPFC/OFC provides a neuroanatomical template for further studies of sex differences in social cognition in males and females with ASD.

### Developmental origins of the cortical sex differences

The surface-based morphometry method used here allowed for a better characterization of the nature of the cortical changes in ASD than previous studies. While the developmental mechanisms leading to altered cortical volume are not well understood, they are known to be related to complex age-dependent cortical maturation and aging processes [26, 27, 95]. Investigating the developmental origins of sex differences in cortical thickness in ASD will require careful analysis of the trajectory of developmental changes from early infancy. Our sample size, despite being the largest of its kind studied to date, did not allow us to examine sex differences in trajectories of cortical changes with age. Identifying sex differences in cortical thickness trajectories in typically developing individuals requires much larger sample sizes and a longitudinal design [96], and this may be true of ASD as well. An important question for future research is whether there are differences in the developmental trajectories of sex differences in STG regions identified in the present study.

In contrast, there is a large body of literature supporting the notion that modified gyrification results from disruptions early in cortical development [24, 25, 23, 22]. Based on the extant data, we suggest that the significant sex-by-diagnosis interaction in gyrification, with selectively reduced gyrification in the vmPFC/OFC of males with ASD, may have its origins in early development. The process of cortical folding starts at 16 weeks post-conception and ends within the first few months of life [64]. Altered patterns of cortical folding are thus assumed to result from adverse events that occur during this period. For instance, premature birth [24, 25], obstetric complications [23], or cardiac surgery during the first months of life [22] are known to affect gyrification. Another prenatal factor that is known to shape the brain of developing fetuses is the level of fetal testosterone [97]. In a brain imaging study of children aged 8 to 11 years, Lombardo and colleagues assessed how variations in levels of fetal testosterone predicted local gray matter volume. Although their study did not distinguish between volume, gyrification, and thickness, they noted that increased fetal testosterone was related to patterns of both increased and decreased gray matter. In particular, the OFC showed a negative correlation between fetal testosterone level and gray matter volume. In light of the recent evidence that fetal steroidogenic activity is elevated in male children who will develop autism later on [7], our finding of altered vmPFC/OFC gyrification in males with ASD supports the idea that elevated fetal testosterone in males with ASD may influence early development of the brain in utero.

### Stability and power analysis

We took advantage of the largest dataset to date provided by the ABIDE Consortium to gain information about the minimal sample size required to observe robust statistical sex differences. Structural imaging studies in ASD have been characterized by a strikingly poor rate of replication, requiring meta-analyses of multiple studies to obtain a clearer picture of the neuroanatomical phenotype in ASD [98, 99, 14]. As such, results based on small samples of affected females might be biased by high inter-individual variability and low power, thereby resulting in inconsistent findings of sex differences in previous studies [12, 13, 11, 10]. To test the hypothesis that sample size has a major impact on the likelihood to observe significant results, we leveraged the large ABIDE dataset to randomly simulate different cohorts with sample sizes ranging from 15 to 50 females with ASD, with their matched counterparts, for a total of 60 to 200 participants. The results of the bootstrap analysis demonstrated that the likelihood of observing significant sex-by-diagnosis interactions, and to a lesser extent for observing a main effect of sex, drastically dropped with decreasing sample size. For instance, with a sample of 30 females with ASD (total *n* = 120), a significant sex-by-diagnosis interaction was only captured in 50 % of the simulated subsamples. The likelihood of observing a sex-by-diagnosis interaction in the vmPFC/OFC cluster increased to 60 % with a sample of 35 females (total *n* = 140), and 90 % for a sample of 45 females with ASD (total *n* = 180). We also investigated this issue by computing post-hoc power based on the estimated effect size identified in our significant clusters. This analysis confirmed that samples of 30 to 55 females with ASD (total *n* = 120–220) are required to observe a significant main effect of sex as well as a sex-by-diagnosis interaction in the same clusters with a power of at least 0.8. Taken together, the simulation analysis and the power computation supports the view that identifying robust and reproducible findings probably requires samples of females with ASD much larger than previously studied.

### Limitations

One of the limitations of our study is that the ABIDE cohort includes mainly individuals with high-functioning ASD (IQ range 61–147). Given the difficulty of acquiring MRIs from lower-functioning children and adolescents, the inclusion of only high-functioning affected individuals is a common limitation of imaging studies in ASD. A second limitation is that we did not have access to fine-grained measures of the clinical and cognitive phenotypes of the individuals with ASD included in the ABIDE cohort. With the available measures, the groups of males and females with ASD did not differ on the severity of autistic symptoms in social interactions, communication, or RRB, limiting our ability to assess whether the observed sex-by-diagnosis interaction has an impact on the observed behavioral phenotype. Finally, to achieve a sample size of 53 females with ASD, we had to merge cerebral MRI acquired at different sites with varying scanning parameters. To minimize the impact of the different sites on quality, we conducted thorough quality control, and matched all the females individually for age and site by “quartet”. Given the dearth of studies examining females with ASD to date, we suggest that our study offers a template for directing future studies examining sex differences in the disorder.

## Conclusion

Using local cortical properties in a unique sample of 210 children, adolescents, and adults, we identified specific neuroanatomical features of typical sex differences that are preserved in individuals with ASD, as well as those that are not. In typically developing individuals, larger cortical volume in the pSTG/PT volume in females has been linked to sex differences in language processing and language acquisition [66–69]. This pattern of sex differences was preserved in individuals with ASD, pointing to a neuroanatomical basis for clinical findings that females with ASD might acquire language faster than affected males [77, 78, 74, 79]. We also observed a significant sex-by-diagnosis interaction, characterized by reduced gyrification of the vmPFC/OFC region in males with ASD. Early prenatal or perinatal disruption in cortical folding development in this cortical region may underlie greater social deficits in males compared to females, with ASD. Future large-scale brain imaging studies including more fine-grained assessments of social and language skills are required to replicate these findings and to better examine the relationship between sex differences in the brain structure and in behavioral and clinical phenotypes. Finally, our bootstrap analyses demonstrated that large sample sizes are required when examining sex differences in neuroanatomical features in ASD. The potential effect of sampling within a highly heterogeneous disorder further stresses the need for a better framework to divide individuals with autism into clinically, etiologically, and neurobiologically homogeneous subgroups, a challenge that requires larger samples than currently available.

## Additional files

Additional file 1: Table S1. Full description of the participants included in the present study. Additional file 2: Figure S1. Comparison of gyrification patterns two-by-two (whole-brain analyses).

## Abbreviations

ASD: autism spectrum disorders
DTI: diffusion tensor imaging
OFC: orbitofrontal cortex
pSTG: posterior superior temporal gyrus
PT: planum temporale
RRBI: repetitive and restricted behaviors and interests
TD: typically developing
vmPFC: ventromedial prefrontal cortex
