# Supplementary material for: Sex differences in cortical volume and gyrification in autism
Source: Mol Autism. 2015 Jul 4;6:42. doi: 10.1186/s13229-015-0035-y (PMC4491212; doi:10.1186/s13229-015-0035-y)
Supplement: Additional file 1: Table S1. — Full description of the participants included in the present study. [file 13229_2015_35_MOESM1_ESM.pdf]

## Additional Table 1: Full description of the participants included in the present study

| | | ASD F | | ASD M | | CTL F | | CTL M | |
|---|---|---|---|---|---|---|---|---|---|
| | Site | ID | Age | ID | Age | ID | Age | ID | Age |
| 1 | CALTECH | 51462 | 20.2 | 51468 | 20.1 | 51479 | 20.0 | 51478 | 19.7 |
| 2 | CALTECH | 51463 | 20.2 | 51465 | 20.2 | 51480 | 20.8 | 51483 | 20.9 |
| 3 | CALTECH | 51471 | 22.4 | 51457 | 22.9 | 51482 | 21.1 | 51488 | 23.3 |
| 4 | CALTECH | 51460 | 34.6 | 51461 | 37.7 | 51493 | 29.2 | 51476 | 39.3 |
| 5 | CMU | 50644 | 19.0 | 50645 | 20.0 | 50661 | 20.0 | 50657 | 21.0 |
| 6 | CMU | 50648 | 31.0 | 50653 | 30.0 | 50656 | 28.0 | 50666 | 31.0 |
| 7 | CMU | 50650 | 31.0 | 50642 | 33.0 | 50669 | 30.0 | 50665 | 33.0 |
| 8 | KKI | 50795 | 8.1 | 50802 | 8.2 | 50784 | 8.1 | 50777 | 8.4 |
| 9 | KKI | 50792 | 8.2 | 50793 | 8.4 | 50790 | 8.8 | 50809 | 8.4 |
| 10 | KKI | 50798 | 10.2 | 50791 | 10.2 | 50812 | 10.0 | 50782 | 10.2 |
| 11 | KKI | 50796 | 12.0 | 50800 | 11.8 | 50810 | 11.9 | 50822 | 12.4 |
| 12 | LEUVEN | 50743 | 14.2 | 50747 | 14.2 | 50723 | 13.8 | 50725 | 14.2 |
| 13 | LEUVEN | 50749 | 15.3 | 50756 | 14.9 | 50735 | 14.3 | 50733 | 14.7 |
| 14 | MAX_MUN | 51606 | 29.0 | 51322 | 29.0 | 51368 | 28.0 | 51339 | 29.0 |
| 15 | MAX_MUN | 51325 | 32.0 | 51320 | 31.0 | 51366 | 32.0 | 51338 | 30.0 |
| 16 | MAX_MUN | 51321 | 45.0 | 51330 | 45.0 | 51372 | 46.0 | 51373 | 46.0 |
| 17 | NYU | 50952 | 8.7 | 51011 | 8.8 | 51040 | 8.5 | 51081 | 8.8 |
| 18 | NYU | 50958 | 10.2 | 50971 | 10.2 | 51044 | 10.9 | 51120 | 10.2 |
| 19 | NYU | 50953 | 11.8 | 51007 | 11.9 | 51046 | 11.6 | 51122 | 11.9 |
| 20 | NYU | 50956 | 14.1 | 50972 | 14.0 | 51051 | 14.1 | 51075 | 14.2 |
| 21 | NYU | 50954 | 14.8 | 50999 | 14.5 | 51053 | 14.4 | 51100 | 14.4 |
| 22 | NYU | 50957 | 14.8 | 50966 | 15.1 | 51054 | 14.4 | 51104 | 15.3 |
| 23 | NYU | 50959 | 22.8 | 51027 | 23.0 | 51059 | 22.8 | 51151 | 23.1 |
| 24 | NYU | 50962 | 24.4 | 51021 | 23.7 | 51061 | 27.0 | 51067 | 23.7 |
| 25 | NYU | 50961 | 25.0 | 51029 | 26.5 | 51062 | 27.8 | 51153 | 26.2 |
| 26 | NYU | 50960 | 38.8 | 51024 | 39.1 | 51063 | 29.1 | 51068 | 31.8 |
| 27 | OLIN | 50127 | 14.0 | 50118 | 15.0 | 50113 | 15.0 | 50116 | 15.0 |
| 28 | OLIN | 50119 | 19.0 | 50130 | 18.0 | 50114 | 20.0 | 50112 | 17.0 |
| 29 | OLIN | 50125 | 24.0 | 50131 | 21.0 | | | 50109 | 20.0 |
| 30 | PITT | 50029 | 11.4 | 50027 | 12.2 | 50036 | 13.3 | 50033 | 12.2 |
| 31 | PITT | 50057 | 12.6 | 50028 | 12.7 | 50038 | 14.0 | 50051 | 12.8 |
| 32 | PITT | 50023 | 12.6 | 50006 | 13.4 | 50049 | 15.8 | 50031 | 12.9 |
| 33 | PITT | 50005 | 13.7 | 50056 | 13.6 | 50059 | 21.2 | 50043 | 13.8 |
| 34 | UCLA | 51228 | 8.4 | 51249 | 8.5 | 51267 | 11.3 | 51278 | 9.2 |
| 35 | UCLA | 51226 | 9.3 | 51295 | 10.0 | 51282 | 12.7 | 51308 | 9.8 |
| 36 | UCLA | 51215 | 11.6 | 51202 | 11.6 | 51303 | 13.0 | 51262 | 11.6 |
| 37 | UCLA | 51219 | 13.9 | 51300 | 14.1 | 51264 | 13.5 | 51266 | 14.0 |
| 38 | UCLA | 51207 | 14.1 | 51297 | 14.3 | 51279 | 13.8 | 51269 | 14.3 |
| 39 | UCLA | 51230 | 17.5 | 51237 | 17.4 | | | 51268 | 17.8 |
| 40 | UM | 50300 | 9.7 | 50307 | 9.7 | 50357 | 9.5 | 50333 | 10.2 |
| 41 | UM | 50302 | 10.4 | 50304 | 11.0 | 50374 | 9.7 | 50334 | 11.0 |
| 42 | UM | 50285 | 12.8 | 50298 | 12.8 | 50338 | 13.5 | 50370 | 12.8 |
| 43 | UM | 50319 | 15.0 | 50282 | 15.2 | 50369 | 14.8 | 50327 | 15.3 |
| 44 | UM | 50276 | 16.8 | 50273 | 16.8 | 50340 | 16.1 | 50351 | 16.8 |
| 45 | UM | 50321 | 18.0 | 50399 | 17.4 | 50341 | 18.2 | 50328 | 17.4 |
| 46 | YALE | 50615 | 8.6 | 50609 | 8.9 | 50576 | 8.4 | 50554 | 8.7 |
| 47 | YALE | 50627 | 9.5 | 50622 | 9.9 | 50565 | 11.0 | 50566 | 9.7 |
| 48 | YALE | 50624 | 11.1 | 50626 | 11.1 | 50558 | 13.8 | 50571 | 11.0 |
| 49 | YALE | 50620 | 12.8 | 50613 | 12.5 | 50563 | 13.9 | 50559 | 12.3 |
| 50 | YALE | 50623 | 13.7 | 50603 | 13.3 | 50557 | 14.3 | 50561 | 13.3 |
| 51 | YALE | 50604 | 14.4 | 50628 | 14.4 | 50555 | 14.4 | 50573 | 14.2 |
| 52 | YALE | 50619 | 16.5 | 50605 | 16.0 | 50572 | 15.8 | 50551 | 15.9 |
| 53 | YALE | 50621 | 16.6 | 50608 | 16.6 | 50569 | 16.7 | 50570 | 16.7 |

The IDs refer to the ones used in the ABIDE description that can be found at http://fcon_1000.projects.nitrc.org/indi/abide/. After quality control, we identified for each female with ASD one ASD male, TD male and TD female with the closest age that was acquired in the same site. Each row in the table details the ID and age of the resulting “quartet”.
