# Supplementary material for: Sex differences in cortical volume and gyrification in autism
Source: Mol Autism. 2015 Jul 4;6:42. doi: 10.1186/s13229-015-0035-y (PMC4491212; doi:10.1186/s13229-015-0035-y)
Supplement: Additional file 2: Figure S1. — Comparison of gyrification patterns two-by-two (whole-brain analyses). [file 13229_2015_35_MOESM2_ESM.pdf]

Supplementary Figure 1:
Comparison of gyrification patterns two-by-two (whole brain analyses)

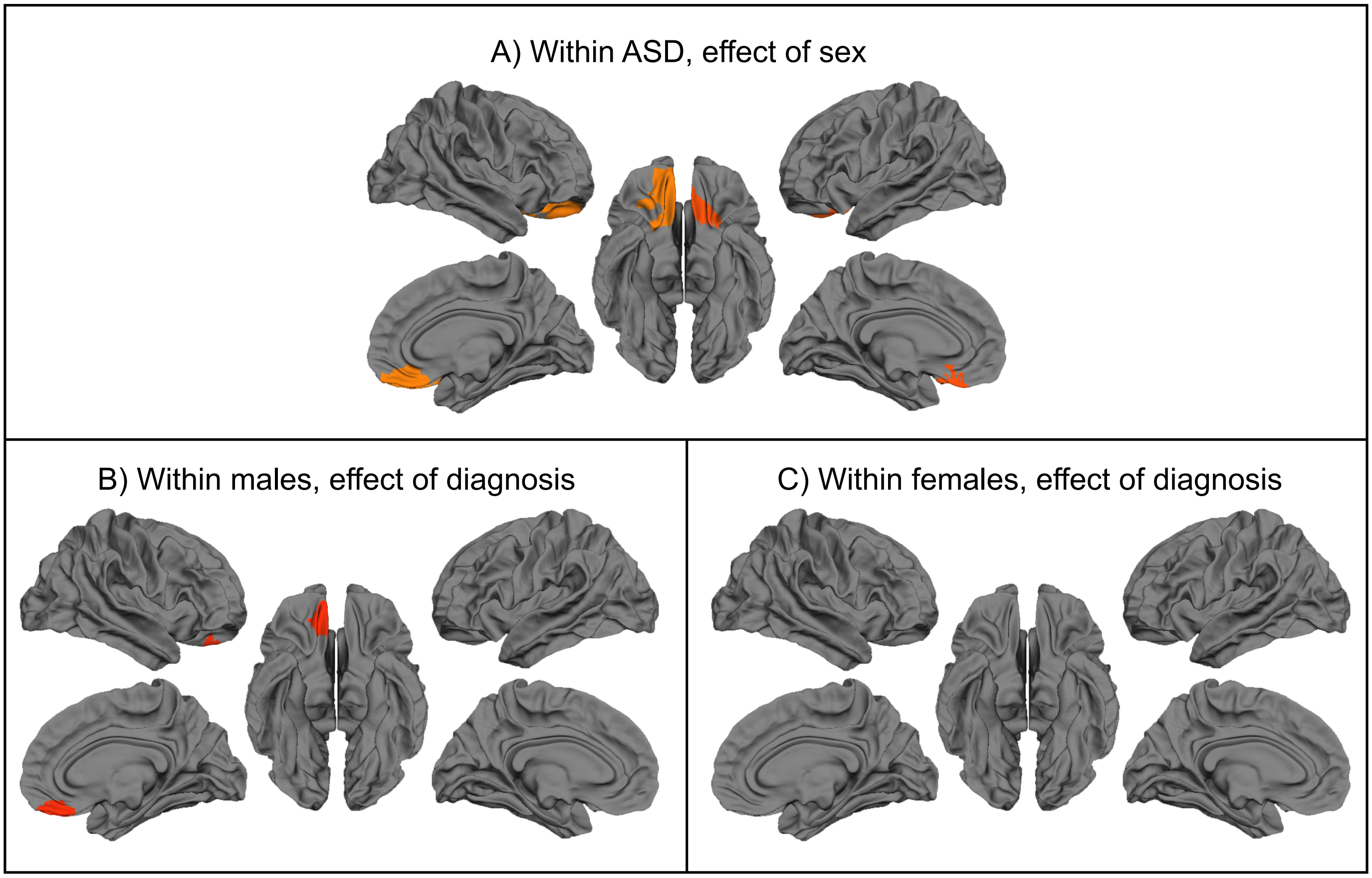

a) Within individuals with ASD, males showed significantly decreased gyrification in the bilateral orbitofrontal / ventromedial region compared to females.

b) Reduced gyrification in the vmPFC/OFC region was observed when comparing ASD males to TD males.

c) No significant differences was found when comparing ASD females and TD females. Overall, the results of whole-brain analyses support a robust decrease in the right vmPFC/OFC gyrification in ASD males compared to both TD males and ASD females.
